# Everybody's Page

**Published:** 1888-06-09

**Authors:** 


### Everybody's Page.

Over-work of the stomach or other parts of the digestive apparatus is a common cause of dyspepsia.

During each day every man, woman, and child consumes, on the average, 8 oz., or ½ lb., of carbon within the animal frame.

In London, in 1858, a young woman of twenty had considerable difficulty in persuading a clergyman to perform the marriage ceremony in consequence of her possessing a bushy beard four inches long.

The average growth of the beard has been computed to be 6½ inches each year. A man of eighty years of age, therefore, who has shaved regularly all his life may be said to have sacrificed to the razor about 35 feet of hair.

After birth, and for some time, growth is very active, and this means a greater requirement for food and a lessened power of resisting unfavourable surroundings. So important is this growth, that it is said to be the cause of the fact that the mortality of male children after birth is greater than that of females.

A dairyman, charged with the usual failing of his fraternity, gave as his excuse that his cows had lately been fed on hay; and the next comer, accused of like unprofessional conduct, and seeing how successful his fellow-sinner's explanation had been, and determined not to be outdone, announced triumphantly that his cows had been fed on straw. The pleading was effectual, and both men got off.

In Egypt the temples of Saturn, and in Greece the Asclepian hospitals, were resorted to by those mentally afflicted, and the treatment there adopted was identical in principle (and perhaps superior in practice) with that of the present day. The directions given by all the classical medical authors, and especially by Hippocrates, who flourished 400 B.C., and by Galen, who practised a century B.C., are of the soundest character.

In the eleventh century, so much did the trade of baker stand in need of regulation, that what was called an assize of bread was instituted. This was followed by the Statute of Assize, and although at first the main object of this statute was not the suppression of adulteration, as time went on the malpractices of bakers became so flagrant that clauses were introduced stating punishment for certain offences. Thus we read in the assize of 1582: "If there be any that by false meanes useth to sell meale; for the first tyme he shall be grievously punished, the second tyme he shall lose his meale, the third tyme he shall foreswere the town, and so likewise the bakers that offend."

"Seven" being the average size of a man's head as measured by his hat, it appears that out of fourteen distinguished persons, two (Lord Chelmsford and Dean Stanley) were below, while other two (Lord Beaconsfield and the Prince of Wales) were exactly up to the average. Of the others, Dickens, Selborne, and Bright required 7⅛; Earl Russell, 7¼; Lord Macaulay, Gladstone, and Thackeray, 7⅝; Louis Philippe, 7¾; and the Archbishop of York, 8 full! Of twenty-three distinguished men whose actual brain-weights are known, four, including the late Professor Hughes Bennet, and Hermann, the philologist, were distinctly below the average, showing, as Dr. Bastian points out in a recent work, that "a well-constituted brain of small dimensions may be capable of doing much better work than many a larger organ whose internal constitution is, from one cause or other, defective." When there is no such defect, however, the big brain, there is every reason to believe, confers an undoubted advantage on its owner.

If the benefits of civilisation are sought to be extended too rapidly, the tendency to consumption is increased.

Among other Irish abuses which might well be remedied or put an end to is the habit of "waking" the dead. Recently a Mrs. Sproule, of Athlone, invited ten friends to "wake" her deceased husband, who had died of fever, and that in spite of a warning from the authorities. Could a better method of spreading infectious diseases possibly be invented?

The influence of the smoke in obscuring the sun is brought out still more markedly if we compare the number of hours during the months when most smoke is produced, viz., the three first and the three last months of the year. London, in November 1882, received only 24 per cent. of the solar rays, whereas Hastings had 41 per cent., and Edinburgh only 19.

In answer to the question why the duration of life should be 100 years in man, and something else in other animals, a law has been enunciated by Buffon, Flourens, and others, that "the duration of life is five times the duration of growth." Thus in man growth is complete about 20, in the horse at 5, in the ox at 4, in the dog at 2. This gives for the duration of life in man 100 years, in the horse 25, the ox 20, the dog 10.

Perambulators are now so thoroughly an institution of our day for the locomotion of young children that it would be vain to attempt to discountenance them. In winter, however, they are often the cause of their occupants catching colds, when used in biting winds, or when allowed to stand while those in charge of them are enjoying a talk with a friend, or when the body and limbs of the child are not abundantly wrapped up.

The street dust of our large towns has been examined and analysed, and has been found to contain fragments of hay and straw, hairs and fibres, and pollen of plants, cotton and woollen filaments, wings and other fragments of insects, spores and germs of organisms, besides fine particles of lime, coal dust, sand, metal, iron, &c. The dust taken from the roofs of houses, and even from the interior rafters, as well as that found on the tops of pillars, has been found to be similar in nature. The dust of our rooms, too, is of the same character, accompanied by minute fibres, hairs, and scales from skin.

The lactometer, an instrument for measuring milk by its weight—it is considerably heavier than water—has been proved to be nearly useless, as the following incident will show: The governors of one of the London poorhouses inserted in their contract specification the stipulation that the milk supplied to the institution should come up to a certain mark on the lactometer, and for a time they got pretty good milk, until, indeed, in an unfortunate moment one ingenious contractor discovered how the milk was tested, and he soon succeeded—though he supplied only very poor milk—in satisfying the requirements of the lactometer, and at the same time in making a very good profit. He first of all partially skimmed the milk—that made it too heavy; the lactometer stood too high, so as an inexpensive and effectual corrective, he added a little water, which, of course, made the milk lighter, and so he continued the addition until he brought the lactometer to the right point exactly, thus fulfilling to a nicety and in the most scientific manner the requirements of the governors. The only way as yet known to test milk accurately is by means of a properly-conducted chemical analysis.

